# Radiation dose escalation can improve local disease control and survival among esophageal cancer patients with large primary tumor volume receiving definitive chemoradiotherapy

**DOI:** 10.1371/journal.pone.0237114

**Published:** 2020-08-06

**Authors:** Forn-Chia Lin, Wei-Lun Chang, Nai-Jung Chiang, Meng-Ying Lin, Ta-Jung Chung, Tzu-Hui Pao, Wu-Wei Lai, Yau‐Lin Tseng, Yi‐Ting Yen, Bor-Shyang Sheu

**Affiliations:** 1 Department of Radiation Oncology, National Cheng Kung University Hospital, College of Medicine, National Cheng Kung University, Tainan, Taiwan; 2 Department of Internal Medicine, National Cheng Kung University Hospital, College of Medicine, National Cheng Kung University, Tainan, Taiwan; 3 National Institute of Cancer Research, National Health Research Institutes, Tainan, Taiwan; 4 Department of Diagnostic Radiology, National Cheng Kung University Hospital, College of Medicine, National Cheng Kung University, Tainan, Taiwan; 5 Department of Surgery, National Cheng Kung University Hospital, College of Medicine, National Cheng Kung University, Tainan, Taiwan; 6 Institute of Clinical Medicine, College of Medicine, Kaohsiung Medical University, Kaohsiung, Taiwan; Chang Gung Memorial Hospital at Linkou, TAIWAN

## Abstract

**Background:**

This study aimed to investigate the correlation between primary tumor volume and cancer failure patterns in esophageal squamous cell carcinoma (ESCC) treated with definitive concurrent chemoradiotherapy (CCRT) and examine whether increasing radiation dose can improve the outcome.

**Methods:**

We retrospectively reviewed 124 patients with stage III ESCC treated by definitive CCRT. The primary tumor volume calculated from the radiotherapy planning computed tomography scans was correlated to treatment response, time to disease progression, and overall survival. We further analyzed whether a higher radiation dose correlated with better disease control and patient survival.

**Results:**

Patients with poor CCRT response had a larger primary tumor volume than those with good response (97.9 *vs* 64.3 cm^3^, *P* = 0.032). The optimal cutoff value to predict CCRT response was 55.3 cm^3^. Large primary tumor volume (≥ 55.3 cm^3^) correlated with shorter time to tumor progression in the esophagus (13.6 *vs* 48.6 months, *P* = 0.033) compared with small tumor volume (< 55.3 cm^3^). For the large esophageal tumors (≥ 55.3 cm^3^), radiation dose > 60 gray significantly prolonged the time to tumor progression in esophagus (20.3 *vs* 10.1 months, *P* = 0.036) and overall survival (12.2 *vs* 8.0 months, *P* = 0.030), compared with dose ≤ 60 gray. In contrast, higher radiation dose did not benefit local disease control or overall survival in the small esophageal tumors (< 55.3 cm^3^).

**Conclusion:**

Large primary tumor volume correlates with poor local control and overall survival in ESCC treated with definitive CCRT. Radiation dose > 60 gray can improve the outcomes in patients with large primary tumor. Further prospective dose escalation trials are warranted.

## Introduction

Esophageal cancer is the sixth leading cause of cancer-related death globally. The most common histological type is squamous cell carcinoma.[1] Definitive concurrent chemoradiotherapy (CCRT) is one of treatment options for locally advanced esophageal squamous cell carcinoma (ESCC).[2–6] As the outcomes of ESCC patients after definitive CCRT was unsatisfactory, it is imperative to identify the prognostic factors and establish improvement strategies.

Large primary tumor volume has been identified as a poor prognostic factor in several solid cancers treated with definitive CCRT, including head and neck cancer, lung cancer, as well as esophageal cancer.[7–9] Identifying the tumor progression pattern (either local or distant control failure) paves the way for better disease control and survival. However, the impact of esophageal tumor volume on disease progression patterns remains to be elucidated in ESCC patients undergoing definitive CCRT.

In this study, we included a single-institution cohort of patients with locally advanced (stage III) ESCC treated by definitive CCRT. We analyzed the correlation of primary tumor volume with the disease failure pattern. Importantly, we examined whether escalated radiation dose could improve the disease control and survival in patients with large primary tumor volume.

## Patients and methods

### Patients and study design

This study was approved by the institutional review board of National Cheng Kung University Hospital. We included all patients with histologically proven ESCC who received treatment in our hospital during 2008 to 2017. Patients' medical records at National Cheng Kung University Hospital from Jan. 1, 2008 to Oct. 31, 2018 were accessed. Data were not anonymized before we accessed them. The pretreatment staging of tumor was conducted by endoscopic ultrasound (EUS) of the esophagus, computed tomography (CT) scan from chest to abdomen, and bone scan. Positron Emission Tomography—Computed Tomography (PET-CT) was performed in cases with indeterminate results of CT or bone scan. After clinical staging, patients received treatment protocol according to the guideline established by our institutional esophageal cancer study group. All patients having clinical stage III ESCC according to the seventh edition of American Joint Committee on Cancer staging system and undergoing definitive CCRT with dose ≥ 50 gray were considered eligible. All patients received regular follow-up with EUS and CT scan to assess treatment response and time to tumor progression. In addition, bone scan, PET-CT, and other exams were performed as clinically indicated. The date of disease progression and patient death were recorded to define the time to disease progression and overall survival which were calculated from diagnosis.

### The protocol of concurrent chemoradiotherapy

All patients received definitive CCRT for esophageal cancer. Radiotherapy was performed with sliding window intensity modulated radiation therapy (IMRT) at fixed gantry angles, as previously described.[10] Briefly, the gross tumor volume (GTV) consisted of GTV of the primary (GTVp) and GTV of lymph nodes (GTVn). The clinical target volume (CTV) 1 included GTVp with a 5-cm craniocaudal and 1-cm radial margin along the esophagus, and GTVn with a 1-cm margin. The CTV 2 included GTVp with a 2-cm craniocaudal and 1-cm radial margin along the esophagus, and GTVn with a 1-cm margin. The planning target volume (PTV) was generated by expanding 1 cm around the GTV and CTV in all directions. Elective nodal irradiation was omitted. CTV 1 and CTV 2 with the relevant PTV were sequentially treated to 36 and 50–50.4 gray, respectively. Thereafter, GTV with the relevant PTV was boosted up to 66–66.6 gray if dose constraints of the organs at risk could be met. Normal tissue-dose constraints included Dmax < 50 gray for spinal cord, V50 < 33% for heart, V20 < 33% for lung, Dmax < 55 gray for stomach, and V35 < 50% for liver.[5, 10] During radiotherapy, chemotherapy was given concurrently with either one of the regimens shown in Table 1.

**Table 1:** The baseline characteristics of the enrolled patients.

Characteristic	n (%)
Age (year), mean ± SD	56.6 ± 10.4
Gender, female: male	5 (4.0): 119 (96.0)
Tumor histology, squamous cell carcinoma	124 (100.0)
Tumor location, upper: middle: lower	30 (24.2): 40 (32.3): 54 (43.5)
Esophageal tumor length (cm)	
Mean ± SD	5.6 ± 2.3
Median (IQR)	5.0 (2.5)
Esophageal tumor volume (cm^3^)	
Mean ± SD	76.6 ± 53.0
Median (IQR)	63.1 (55.2)
AJCC stage^〒^	
T2: T3: T4	8 (6.5): 85 (68.5): 31 (25.0)
N1: N2: N3	6 (4.8): 50 (40.3): 68 (54.8)
IIIA: IIIB: IIIC	8 (6.5): 40 (32.3): 76 (61.3)
ECOG performance status, 0: 1: 2	16 (12.9): 86 (69.4): 22 (17.7)
Radiation dose (gray)	
Median (IQR)	61.2 (16.2)
≤ 60: > 60	51 (41.1): 73 (58.9)
Chemotherapy regimen	
Platinum-fluoropyrimidine	112 (90.3)
Taxane-based	8 (6.5)
Others	4 (3.2)

^〒^Clinical stage was classified according to the American Joint Committee on Cancer (AJCC) tumor-node-metastasis (TNM) classification, 7^th^ ed.

### Evaluation of esophageal tumor volume

**Fig 1:**
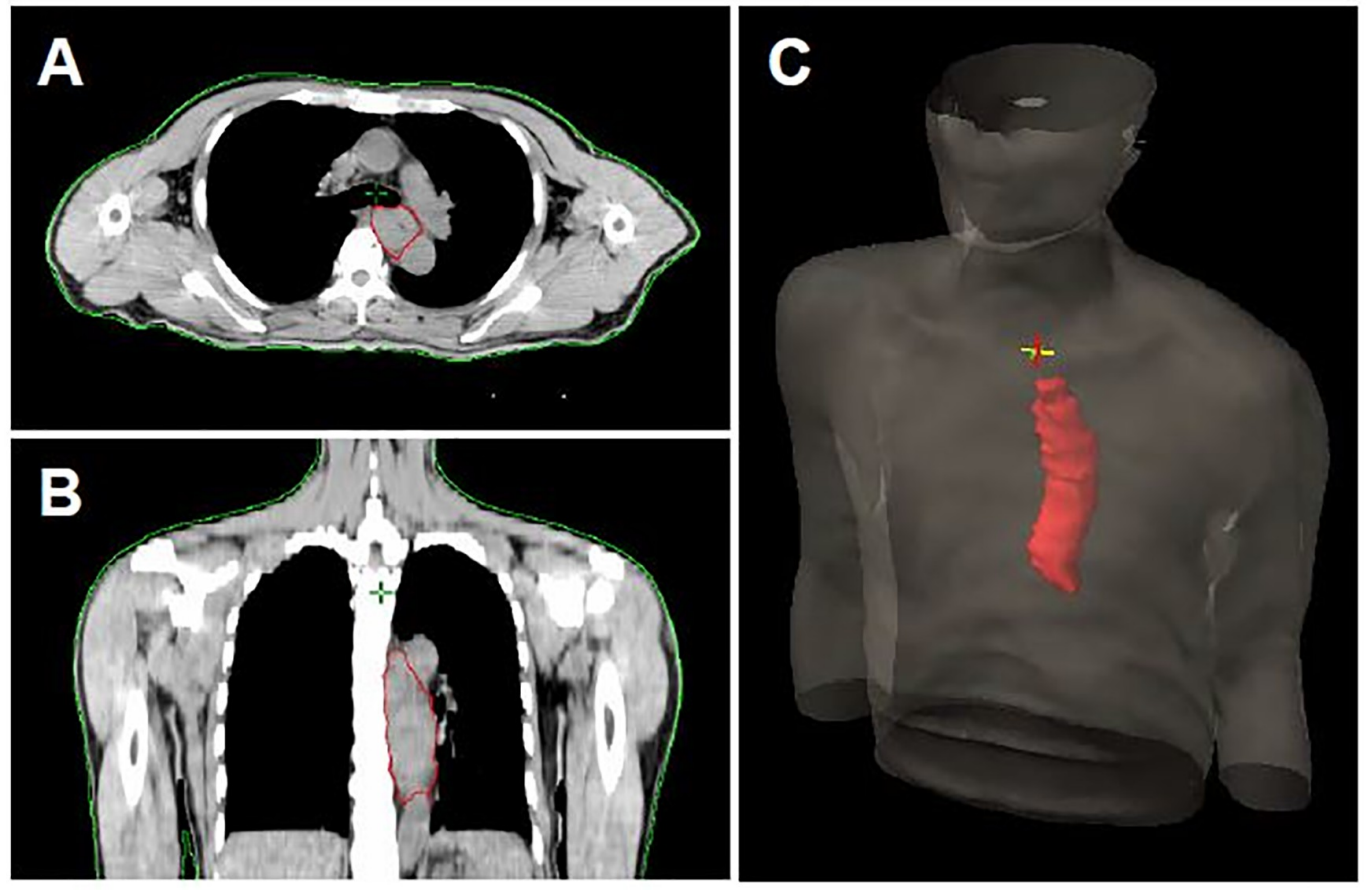
The representative images of primary tumor volume calculation. (A) The cross-sectional area of tumor was circumscribed by red line. (B) The sagittal area of tumor was circumscribed by red line. (C) The 3D construction of primary tumor volume.

The simulation CT scan was acquired at 5mm slice thickness and transferred to Eclipse treatment planning system (Varian Medical Systems). We determined the upper and lower border of esophageal tumor according to the increased esophageal wall thickness shown on CT scan and the tumor location observed on endoscopy and EUS. The esophagus between the upper and lower tumor borders was delineated on each relevant slice of the planning CT scan and defined as the primary tumor. The esophageal tumor volume (in cubic centimeters) was then calculated using the volume computation function integrated into the Varian Eclipse system. (Fig 1).

### Evaluation of treatment response

After completion of 36-gray radiotherapy, a EUS exam was conducted to assess the treatment response.[11] Briefly, the esophageal mucosa and intraluminal lesion were first inspected by general upper gastrointestinal scope. Then, a EUS miniprobe (2.5 mm in diameter) was inserted into esophageal lumen via the working channel to scan the previous tumor area. The maximal post-CCRT esophageal wall thickness was recorded, and compared with baseline data to define the treatment response. A good treatment response was defined as a reduction in tumor thickness by ≥ 30%. A poor treatment response was defined as a reduction in tumor thickness by < 30%.[12]

### Statistical analysis

The analyses were performed using SPSS 22.0 (SPSS, Inc., Chicago, IL, USA). The comparison of esophageal tumor volume between good versus poor CCRT responder was conducted by independent t-test. Receiver operating characteristic (ROC) curve was used to select the optimal cutoff value of esophageal tumor volume to predict CCRT response. The time to tumor progression and overall survival were estimated with Kaplan-Meier method and compared between groups by log-rank test. In multivariate analysis of factors correlated with overall survival, Cox regression analysis was used. Only variables with *P* value less than 0.2 in the univariate analysis were included in the multivariate analysis. The ‘enter’ method was used when conducting multivariate analysis. All tests were 2-tailed with a *P* value less than 0.05 taken as significant.

## Results

### Characteristics of the enrolled patients

Table 1 showed the baseline characteristics of the enrolled 5 female and 119 male patients. They were mainly middle-aged male, with a mean primary tumor volume of 76.6 cm^3^ (median 63.1 cm^3^). Most patients (93.6%) were stage IIIB and IIIC. The median radiotherapy dose was 61.2 gray. Depending on compliance with constraints of the organs at risk, 51 (41.1%) patients had 60 gray or less while the remaining had more than 60 gray. Platinum plus fluoropyrimidine-based combination regimens were utilized in 112 (90.3%) patients. Monthly and weekly combination regimens were administered concurrently with radiotherapy for 2 cycles and 6–8 cycles, respectively. Taxane plus platinum regimens were given biweekly for 3–4 cycles.

### Poor CCRT response correlated with large baseline primary tumor volume

**Fig 2:**
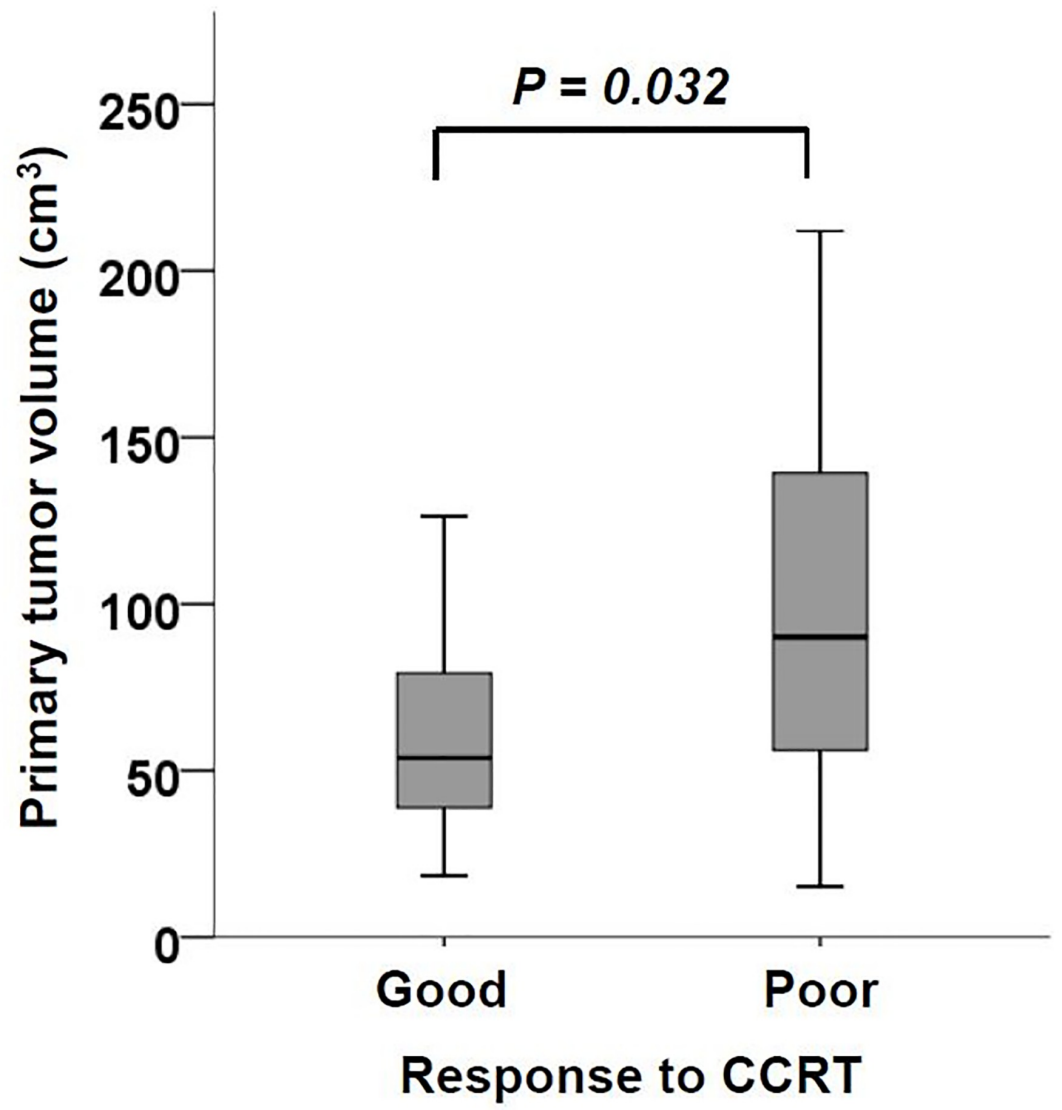
The difference of esophageal tumor volume was shown between good and poor CCRT responders. Patients with poor response to CCRT (tumor thickness reduction < 30%) had significantly larger esophageal tumor volume than those with good response (tumor thickness reduction ≥ 30%).

Fig 2 showed the baseline primary tumor volume distribution in patients with good (≥ 30% tumor thickness reduction) or poor (< 30% tumor thickness reduction) response to CCRT. Patients with poor CCRT response had a larger primary tumor volume than those with good CCRT response (mean ± SD, 97.9 ± 58.9 *vs* 64.3 ± 39.7 cm^3^, *P* = 0.032). The ROC curve set the optimal cutoff value to predict CCRT response as 55.3 cm^3^ (S1 Fig).

### Disease progression patterns

We next investigated the first disease progression site in ESCC patients after CCRT. The median follow-up time was 49.0 months for survivors. As shown in Table 2, the first disease progression site was identified at the esophagus in 41 (33.1%) and regional lymph node in 32 (25.8%) patients. In addition, 44 (35.5%) patients experienced their first progression over distant metastatic sites, including lung in 24, liver in 13, bone in 5, non-regional lymph node in 5, and pleura in 2 patients. Thirty-five (28.2%) patients were alive without disease progression at last follow-up.

**Table 2:** The disease progression pattern in ESCC treated by definitive CCRT.

First disease progression site	n (%)
**No disease progression**	35 (28.2)
**Distant failure only**	
Distant site	32 (25.8)
**Locoregional failure only**	
Esophagus	22 (17.7)
Esophagus + Regional LN^〒^	14 (11.3)
Regional LN	9 (7.3)
**Both locoregional and distant failure**	
Regional LN + Distant site	7 (5.6)
Esophagus + Distant site	3 (2.4)
Esophagus + Regional LN + Distant site	2 (1.6)

^〒^LN: lymph nodes

### Large primary tumor volume correlated with early disease progression in the esophagus, but not regional lymph node or distant sites

**Fig 3:**
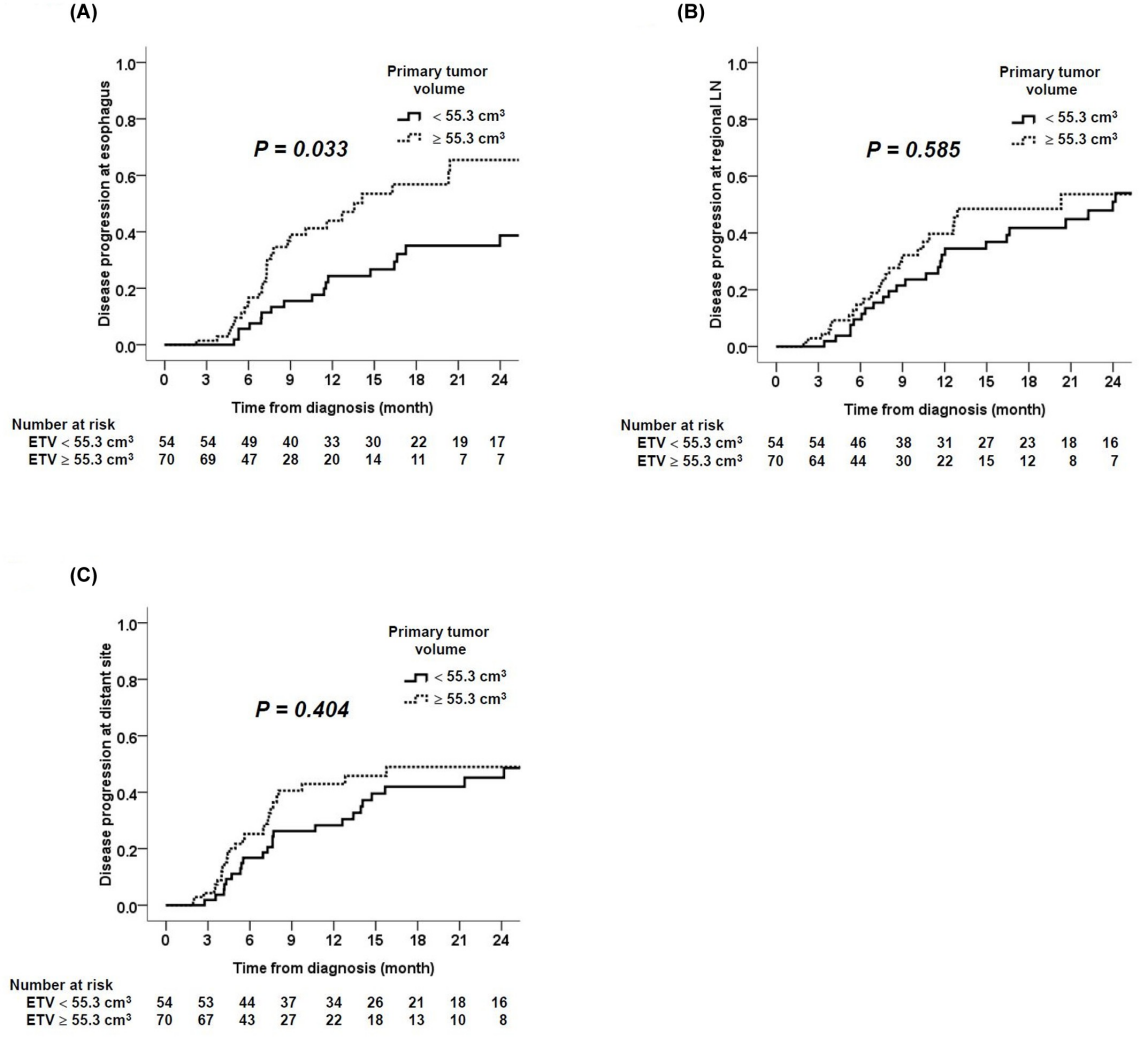
The difference of time to disease progression was shown between patients with different esophageal tumor volumes. (A) Time to disease progression at esophagus was significantly shorter in patients with larger esophageal tumor volume. (B & C) Time to disease progression at regional lymph nodes or other distant sites was similar between patients with different esophageal tumor volumes.

We further analyzed whether tumor volume was related with patterns of tumor progression. Interestingly, patients with large primary tumor volume (≥ 55.3 cm^3^) had significantly earlier disease progression in the esophagus than those with small primary tumor volume (< 55.3 cm^3^, Fig 3A). The median time to progression in the esophagus was 13.6 and 48.6 months in patients with large and small tumor volume, respectively (*P* = 0.033). In contrast, there was no significant difference in the time to progression at regional lymph nodes or distant sites between patients with different esophageal tumor volumes (Fig 3B & 3C).

### High radiation dose improved local disease control of large esophageal tumors

**Fig 4:**
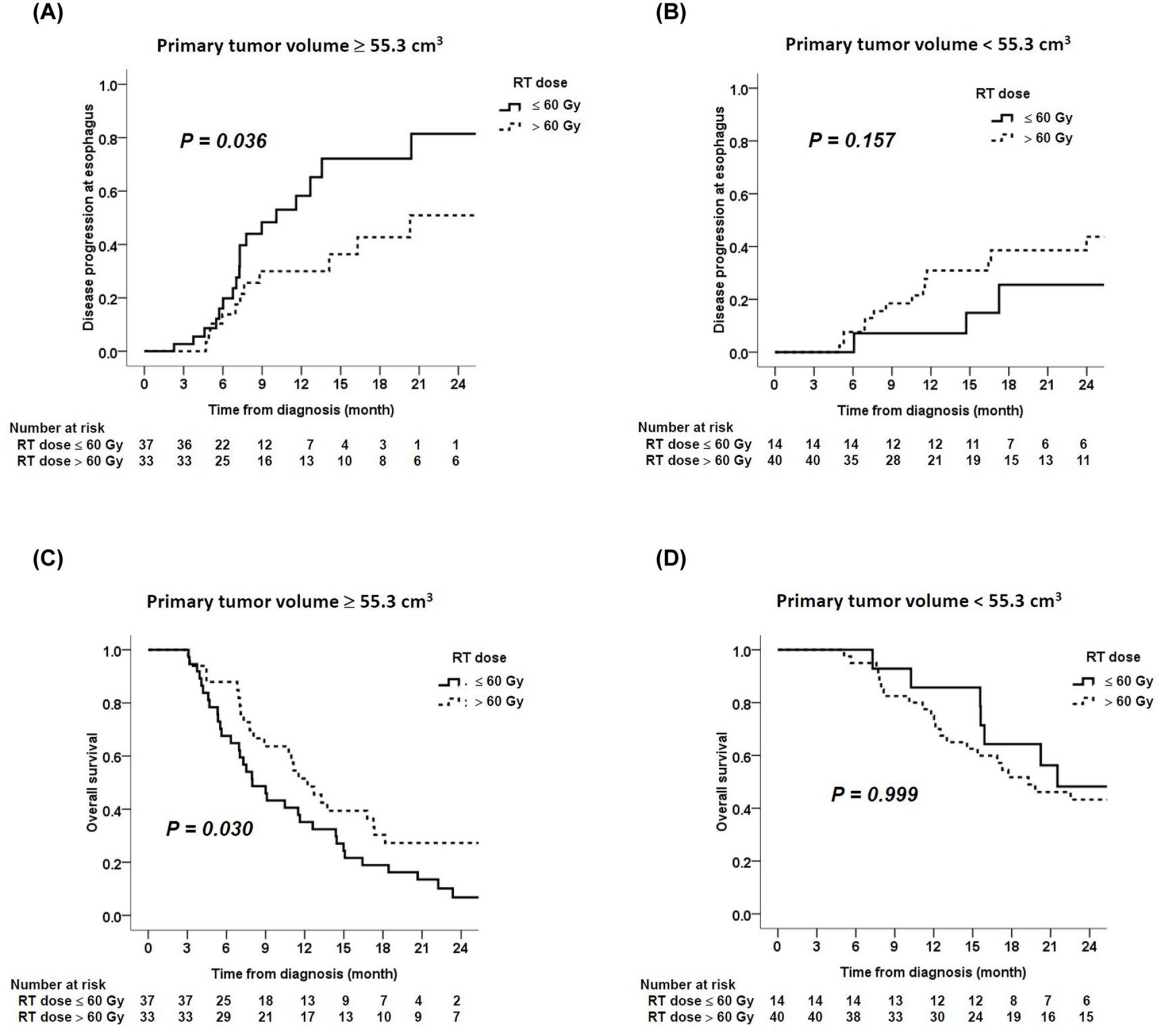
The effect of increasing radiation dose on tumor control according to tumor volume. A higher radiation dose (> 60 gray) delayed time to esophageal failure in patients with esophageal tumor ≥ 55.3 cm^3^ (A), but not in those with esophageal tumor < 55.3 cm^3^ (B). In patients with esophageal tumor volume ≥ 55.3 cm^3^, a higher radiation dose (> 60 gray) improved overall survival (C). However, in patients with esophageal tumor volume < 55.3 cm^3^, a higher radiation dose (> 60 gray) was not shown to have survival benefit (D).

We next investigated whether increasing radiation dose can improve local disease control for large esophageal tumors. In patients with large esophageal tumor (≥ 55.3 cm^3^), radiation dose more than 60 gray prolonged the time to progression in the esophagus compared to dose of 60 gray or less (Fig 4A). The median time to progression in the esophagus was 20.3 versus 10.1 months (*P* = 0.036), respectively. In contrast, increasing the radiation dose did not benefit the local disease control for patients with small esophageal tumor (< 55.3 cm^3^) (Fig 4B). In concordance with local disease control, radiation dose more than 60 gray increased the overall survival time in patients with large esophageal tumor (12.2 versus 8.0 months, *P* = 0.030, Fig 4C), but not in those with small esophageal tumor (Fig 4D).

In multivariate Cox regression analysis (Table 3), esophageal tumor volume and tumor location were two independent factors related with overall survival in stage III ESCC patients receiving definitive CCRT. The data suggested that a high radiation dose would not benefit to all ESCC patients receiving definitive CCRT. And only patients with large esophageal tumor volume would benefit from high radiation dose (Fig 4C & 4D).

**Table 3:** Multivariate Cox regression of factors related with overall survival.

		Single variate		Multivariate	
Variate	Case no.	HR (95% CI)	*P*	HR (95% CI)	*P*
Age (year)					
≤ 56	61	1		1	
> 56	63	1.45 (0.99–2.13)	0.059	1.46 (0.97–2.19)	0.070
Gender					
Female	5	1		-	
Male	119	1.28 (0.47–3.49)	0.626	-	-
Esophageal tumor volume (cm^3^)					
< 55.3	54	1		1	
≥ 55.3	70	2.01 (1.36–2.99)	0.001	2.16 (1.43–3.26)	< 0.001
Tumor location					
Upper	30	1		1	
Middle	40	2.54 (1.45–4.47)	0.001	2.24 (1.27–3.96)	0.006
Lower	54	2.20 (1.29–3.75)	0.004	1.88 (1.08–3.27)	0.026
TNM stage					
IIIA	8	1		-	
IIIB	40	0.64 (0.28–1.46)	0.286	-	-
IIIC	76	1.14 (0.52–2.49)	0.746	-	-
Radiation dose (gray)					
≤ 60	51	1		1	
> 60	73	0.61 (0.41–0.90)	0.013	0.77 (0.51–1.17)	0.220

### Toxicity

Esophageal perforation was identified in 16 (12.9%) patients, resulting in esophago-bronchial fistula in 2, esophago-pleural fistula in 5, esophago-tracheal fistula in 6, esophago-pulmonary fistula in 1, and esophago-aortic fistula in 2 patients. The cumulative incidence of esophageal perforation was not significantly different between patients with radiation dose > 60 and ≤ 60 gray (*P* = 0.080 by log-rank test, S2 Fig). In addition, no patient experienced grade 3 or higher radiation pneumonitis.

## Discussion

The present study analyzed 124 patients with stage III ESCC treated with definitive CCRT. We found large baseline primary tumor volume (≥ 55.3 cm^3^) correlated with poor treatment response, local disease control, and overall survival. Importantly, we showed that radiation dose more than 60 gray significantly improved local disease control and overall survival in patients with large esophageal tumor (≥ 55.3 cm^3^).

Although previous studies had proposed primary tumor volume as an important prognostic factor of esophageal cancer which correlated with shorter overall survival,[9, 13, 14] it is not known whether large primary tumor volume leads to more tumor metastasis or poor CCRT response. We found that ESCC that responded poorly to CCRT had large primary tumor volume than that responded well (Fig 2). In addition, ESCC patients with large primary volume demonstrated more disease control failure in the esophagus, but not in the lymph node or distant sites (Fig 3). These data collectively suggested that poor local response to radiotherapy was a major mechanism of poor prognosis in ESCC patients with large primary tumor volume. In addition, it implied that higher radiation dose may improve local disease control. Previous studies also showed that large primary tumor volume was a predictor of poor CCRT response in head and neck cancer and non-small cell lung cancer.[7, 8, 15] To the best of our knowledge, we are the first to demonstrate this association in ESCC.

In recently published ARTDECO study, there was a trend toward better locoregional progression-free survival at 3 years among patients receiving integrated boost to the primary tumor. However, escalated radiation dose resulted in numerical increase in toxicity but did not improve the overall survival.[16] In line with the ARTDECO trial, the present study did not observe the benefit in local disease control or survival from increasing radiation dose in our whole cohort. Interestingly, we found that increasing radiation dose to more than 60 gray in patients with large primary tumor volume (≥ 55.3 cm^3^) can improve local disease control and overall survival (Fig 4). Although the dose escalation strategy according to tumor size has not been established in ESCC,[2, 3, 16–18] it had been proposed in lung cancer to improve local disease control.[7] Our data indicated a beneficial effect of higher radiation dose in the large ESCC treated by definitive CCRT and suggested to select patients with consideration of tumor size in the future dose escalation trial for esophageal cancer.

The median overall survival in our study cohort was 14.39 months, which was inferior to 19.3 months of the CCRT arm in FFCD 9102 trial. In FFCD 9102 trial, they only enrolled patients with resectable tumors with good response to the first course of CCRT.[3] But our study did not exclude patients with poor response to CCRT. Therefore, the inferior overall survival of our cohort could be realized. It should be noted that some patients with small esophageal tumor still had early tumor progression despite a higher radiotherapy dose (Fig 4B). This data suggests that the other biological factors may be involved in poor CCRT response which cannot be overcome by increasing radiotherapy dose, such as epigenetic dysregulation.[19]

Our study is limited by retrospective analysis and carries with it all of the biases inherent in such a design. Further prospective dose escalation trials in ESCC patients with large primary tumor volume are warranted. Moreover, we used EUS to assess treatment response by measuring tumor thickness reduction percentage. A more precise evaluation of treatment response may be achieved by 3D measurements of tumor volume changes, such as PET-CT scan.[20]

## Conclusions

This study showed ESCC patients with large primary tumor volume had poorer CCRT response and local disease control. Increasing radiotherapy dose to more than 60 gray could improve local disease control and prolong overall survival in ESCC patients with large primary tumor volume.

## Supporting information

S1 Fig (JPG)

S2 Fig (JPG)
