# Supplementary figures and images for: Radiation dose escalation can improve local disease control and survival among esophageal cancer patients with large primary tumor volume receiving definitive chemoradiotherapy
Source: PLoS One. 2020 Aug 6;15(8):e0237114. doi: 10.1371/journal.pone.0237114 (PMC7410311; doi:10.1371/journal.pone.0237114)

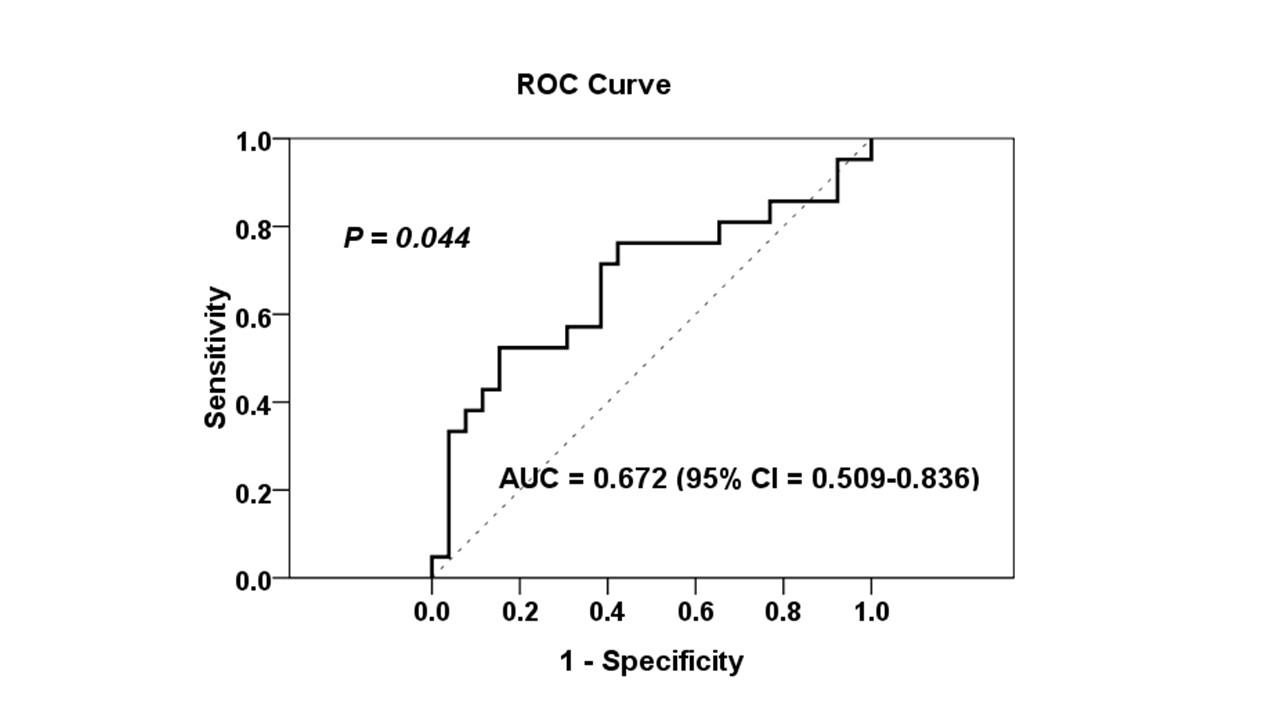

Supplement: S1 Fig — (JPG) [file pone.0237114.s001.jpg]

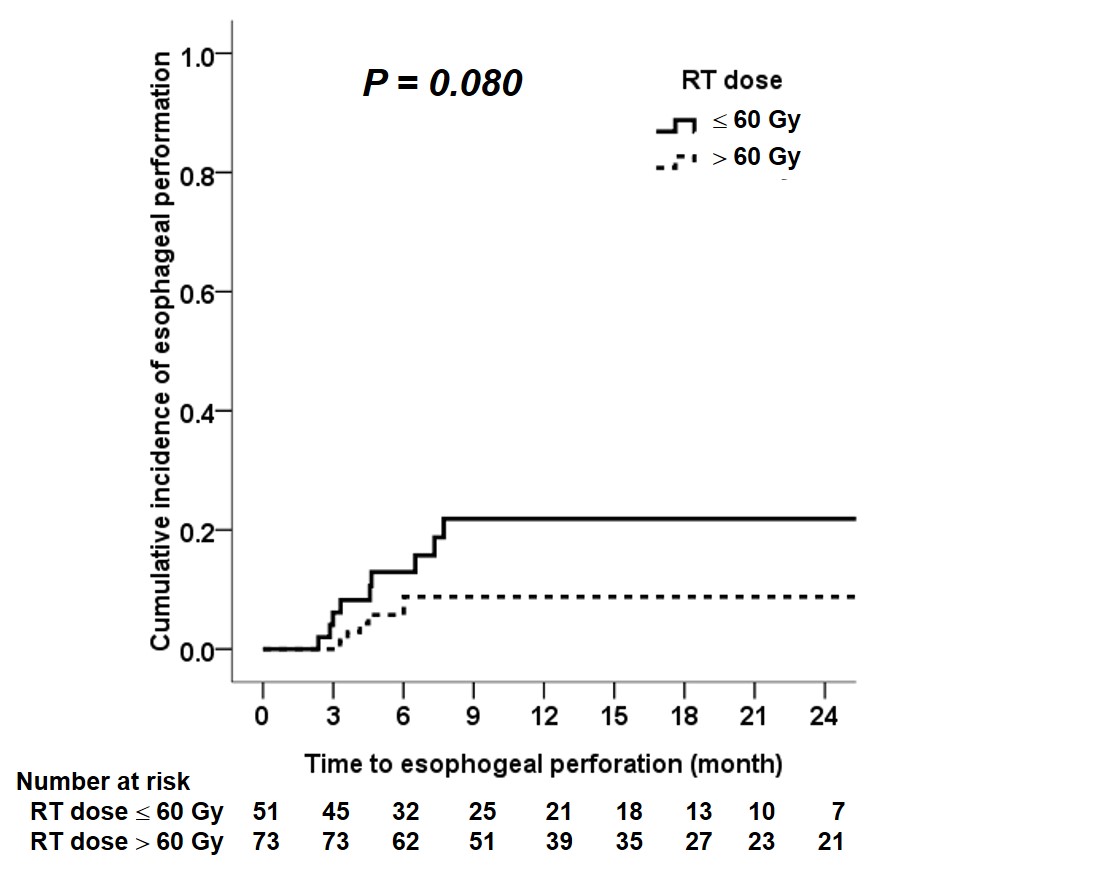

Supplement: S2 Fig — (JPG) [file pone.0237114.s002.jpg]
